# The use of the Gail model, body mass index and SNPs to predict breast cancer among women with abnormal (BI-RADS 4) mammograms

**DOI:** 10.1186/s13058-014-0509-4

**Published:** 2015-01-08

**Authors:** Anne Marie McCarthy, Brad Keller, Despina Kontos, Leigh Boghossian, Erin McGuire, Mirar Bristol, Jinbo Chen, Susan Domchek, Katrina Armstrong

**Affiliations:** Department of Medicine, Massachusetts General Hospital, 50 Staniford Street, 940F, Boston, MA 02114 USA; Department of Radiology, University of Pennsylvania, Philadelphia, PA USA; Abramson Cancer Center, University of Pennsylvania, Philadelphia, PA USA; Department of General Internal Medicine, University of Pennsylvania, Philadelphia, PA USA; Department of Clinical Epidemiology and Biostatistics, University of Pennsylvania, Philadelphia, PA USA

## Abstract

**Introduction:**

Mammography screening results in a significant number of false-positives. The use of pretest breast cancer risk factors to guide follow-up of abnormal mammograms could improve the positive predictive value of screening. We evaluated the use of the Gail model, body mass index (BMI), and genetic markers to predict cancer diagnosis among women with abnormal mammograms. We also examined the extent to which pretest risk factors could reclassify women without cancer below the biopsy threshold.

**Methods:**

We recruited a prospective cohort of women referred for biopsy with abnormal (BI-RADS 4) mammograms according to the American College of Radiology’s Breast Imaging-Reporting and Data System (BI-RADS). Breast cancer risk factors were assessed prior to biopsy. A validated panel of 12 single-nucleotide polymorphisms (SNPs) associated with breast cancer were measured. Logistic regression was used to assess the association of Gail risk factors, BMI and SNPs with cancer diagnosis (invasive or ductal carcinoma *in situ*). Model discrimination was assessed using the area under the receiver operating characteristic curve, and calibration was assessed using the Hosmer-Lemeshow goodness-of-fit test. The distribution of predicted probabilities of a cancer diagnosis were compared for women with or without breast cancer.

**Results:**

In the multivariate model, age (odds ratio (OR) = 1.05; 95% confidence interval (CI), 1.03 to 1.08; *P* < 0.001), SNP panel relative risk (OR = 2.30; 95% CI, 1.06 to 4.99, *P* = 0.035) and BMI (≥30 kg/m^2^ versus <25 kg/m^2^; OR = 2.20; 95% CI, 1.05 to 4.58; *P* = 0.036) were significantly associated with breast cancer diagnosis. Older women were more likely than younger women to be diagnosed with breast cancer. The SNP panel relative risk remained strongly associated with breast cancer diagnosis after multivariable adjustment. Higher BMI was also strongly associated with increased odds of a breast cancer diagnosis. Obese women (OR = 2.20; 95% CI, 1.05 to 4.58; *P* = 0.036) had more than twice the odds of cancer diagnosis compared to women with a BMI <25 kg/m^2^. The SNP panel appeared to have predictive ability among both white and black women.

**Conclusions:**

Breast cancer risk factors, including BMI and genetic markers, are predictive of cancer diagnosis among women with BI-RADS 4 mammograms. Using pretest risk factors to guide follow-up of abnormal mammograms could reduce the burden of false-positive mammograms.

**Electronic supplementary material:**

The online version of this article (doi:10.1186/s13058-014-0509-4) contains supplementary material, which is available to authorized users.

## Introduction

Though mammography screening reduces breast cancer mortality, it is imperfect like all screening tests. The high burden of false-positive tests relative to the number of cancers detected has contributed to controversy about the routine use of mammography screening among women ages 40 to 50, as well as about biennial rather than annual screening [1]. After 10 years of annual mammography screening beginning at age 40, over 60% of women will have a false-positive result and 7% to 9% will have a biopsy [2]. False-positive mammograms can result in inconvenience, pain and anxiety for patients, as well as increased costs [3,4].

Using pretest probability of disease can improve the positive predictive value of a screening test. However, this approach requires the ability to accurately determine an individual’s risk of disease. The Breast Cancer Risk Assessment Tool (BCRAT), or Gail model, uses age, family history of breast cancer, reproductive history and history of breast biopsy or atypical hyperplasia to estimate a woman’s 5-year or lifetime risk of breast cancer [5]. Although the model is well calibrated, its discriminatory accuracy is modest [6]. Additional risk factors, such as genetic markers [7-14] and body mass index (BMI) [15-19], have been shown to moderately improve breast cancer risk prediction.

Although many studies have focused on predicting cancer risk in the general population, few have employed risk prediction models to improve decisions about follow-up of abnormal mammograms. Current standards in the United States recommend biopsy of a mammographic abnormality if the radiologist deems the probability of cancer diagnosis to be at least 2% [20-22]. Mammogram results are reported using the American College of Radiology (ACR) Breast Imaging-Reporting and Data System (BI-RADS), which includes six result categories, each tied to follow-up recommendations [20]. The BI-RADS 4 category indicates the presence of a suspicious abnormality that should be followed up with a biopsy. However, the 1-year probability of breast cancer for women with a BI-RADS 4 mammogram is 15% to 30% on average [20,22-29]; therefore, the majority of biopsies of BI-RADS 4 abnormalities are benign. Furthermore, the likelihood of cancer diagnosis varies widely within the BI-RADS 4 category, leading to the subdivision of the category into BI-RADS 4A (2% to 9% risk of malignancy), BI-RADS 4B (10% to 49% risk of malignancy) and BI-RADS 4C (50% to 94% risk of malignancy) [22]. A small pilot study suggested that an experienced radiologist using this substratification scheme could increase the threshold for the biopsy decision without missing invasive cancers [30]. In addition, a recent modeling study suggested that the addition of pretest breast cancer risk factors, including genetic markers, could change biopsy decisions for a small proportion of women with abnormal mammograms [31]. Greater ability to predict cancer outcomes in women with BI-RADS 4 mammograms could reduce the burden of false-positive tests from mammography.

In this study, we assessed the usefulness of the Gail model, BMI and a panel of 12 single-nucleotide polymorphisms (SNPs) to predict cancer diagnosis among women with BI-RADS 4 mammograms. We then evaluated the extent to which these factors could improve decisions about biopsy among this group by reclassifying women without cancer below the biopsy threshold.

## Methods

### Participants

Women referred for breast biopsies at the Hospital of the University of Pennsylvania following a BI-RADS 4 mammogram between January 2010 and April 2012 were invited to participate in the study. Women were excluded if they were younger than 20 years old, had a personal history of breast or ovarian cancer, mantle radiation or known *BRCA1/2* mutation. Women who consented provided a buccal swab for DNA testing prior to their biopsy appointment. Three hundred sixty-three women were enrolled. An additional 119 women with a BI-RADS 4 mammograms from a previous study in which breast imaging modalities were compared at the same institution were also included (2002 to 2006; National Institutes of Health grant P01 CA85484; Principal Investigator: M Schnall). Participants in the breast imaging study were enrolled between July 2003 and August 2007. A blood sample from each patient was collected and stored, which was used for genetic analysis. Of the total sample, five patients were missing follow-up information, eleven had data on fewer than nine SNP markers and two had nonbreast malignancies (tubular adenoma, B-cell lymphoma in the breast). These participants were excluded, resulting in a total population of 464 for analysis. Both studies were approved by the University of Pennsylvania Institutional Review Board, and written informed consent was obtained from each study participant.

### Risk factors

Participants completed a health history questionnaire, including information on race, age at menarche, age at first live birth, number of biopsies, presence of atypical hyperplasia and family history of breast and ovarian cancer. Using the BCRAT, we estimated the 5-year absolute risk and relative risk (RRs) of breast cancer using source code version 3.0 from the National Cancer Institute website [32]. BMI was calculated by using the patient’s self-reported weight and height at the time of recruitment, or it was extracted from medical record data prior to recruitment.

### Single-nucleotide polymorphism panel

Buccal swabs (*N* = 347) or blood samples (*N* = 117) were sent to deCODE genetics (Reykjavik, Iceland) for analysis using Illumina Infinium II whole-genome genotyping (Illumina, San Diego, CA, USA). The deCODE genetics SNP assay included 12 loci that have consistently been associated with breast cancer risk: 2q35 (rs13387042), MRPS30 (rs4415084), FGFR2 (rs1219648), TNRC9/TOX3 (rs3803662), 8q24 (rs13281615), LSP1 (rs3817198), 5q11 (rs889312), NEK10 (rs4973768), 1p11 (rs11249433), RAD51L1 (rs999737), COX11 (rs6504950) and CASP8 (rs1045485) [33-40]. The call rate was 99.8%. The deCODE BreastCancer™ test uses individual allele effect sizes for the 12 SNPs to create a RR estimate for each genotype. For each participant, a combined RR estimate for the 12-SNP panel was calculated by multiplying the RR estimates for all SNPs as described previously [11]. Expected and observed allele frequencies and homozygote odds ratios (ORs) for risk alleles are included in Additional file 1. The combined SNP panel RR estimate has been shown to be independent of BCRAT factors [11].

### Statistical analysis

The results of the BIRADS 4 biopsies were obtained from pathology records. Logistic regression was used to assess the association of Gail risk factors, BMI and SNP panel RR with cancer diagnosis (invasive or ductal carcinoma *in situ* (DCIS)). First, each predictor was tested in an age-adjusted model. SNP panel RRs were examined as a log-transformed continuous variables and as categorized RRs <1.00, 1.01 to 1.49 and ≥1.50. The Gail RR was tested as a log-transformed continuous variable. Gail absolute 5-year risk estimate was categorized as <1.67% and ≥1.67%, as these cutoffs have been widely used to denote high risk of breast cancer, as well as for the use of chemopreventive drugs [41,42]. BMI data were missing in 17% of participants, and therefore BMI was entered into models, including a category for missing data, as follows: <25 kg/m^2^, 25 to 29.9 kg/m^2^, ≥30 kg/m^2^ and missing. The multivariate logistic regression model included log-transformed SNP panel RR, all Gail risk factors (age, race/ethnicity, age at menarche, age at first live birth, first-degree family history of breast cancer, breast biopsy, atypical hyperplasia) and BMI. We also examined the predictive ability of the various risk factors. Model calibration was assessed using the Hosmer-Lemeshow goodness-of-fit test to compare observed and predicted outcomes within deciles of predicted risk for each model [43]. Discriminatory accuracy was assessed by calculating area under the receiver operating characteristic curve (AUC). DeLong’s test was used to compare AUCs for various models. In our analysis, the model incorporating age and the Gail RR had poor calibration. The original Gail model incorporated 5-year intervals of age, but we entered age as a continuous predictor to minimize the number of predictors in our models. Because of the poor calibration of the age plus Gail RR model, we also examined a model that entered all Gail risk factors individually, and this model was better calibrated to our data. In addition, we performed tenfold cross-validation of the prediction models in the total study population. Finally, we estimated the predicted probability of cancer using the multivariate model and assessed reclassification below several risk thresholds (2%, 3%, 5% and 10%) for cancer cases and noncancer cases. Statistical analyses were performed using SAS 9.3 (SAS Institute, Cary, NC, USA) and Stata/IC 12 (College Station, TX, USA) software.

## Results

The mean age of study participants was 48.7 years (SD, 13.2), and approximately one-half of the study population was over age 50 (Table 1). Over 30% of participants were black or African American. The mean 5-year breast cancer risk estimate derived by using the BCRAT was 1.54, and 33% of participants had a 5-year risk estimate of 1.67% or greater. The mean SNP panel RR was 1.22 (SD, 0.44). Over one-fourth of participants had a SNP panel RR estimate of 1.50 or greater, indicating their risk of breast cancer was 50% greater than that of the general population. Of the 464 participants, 74 women (16%) were diagnosed with cancer, 33 (7%) with DCIS and 41 (9%) with invasive cancer.

**Table 1:** **Characteristics of BIRADS 4 cohort, all ages,** ***N*** **= 464** ^**a**^

**Characteristics**	**Data**	
Age, yr, mean ± SD (range)	48.7 ± 13.2	(20 to 86)
Age, yr, categories		
<35	73	15.7
35 to 40	41	8.8
40 to 49	114	24.6
50 to 59	146	31.5
60+	90	19.4
Race/ethnicity		
White	277	59.7
African American/black	145	31.3
Hispanic	9	1.9
Asian	16	3.5
Other	17	3.7
Age at menarche, yr		
<11	90	19.4
12 to 13	200	43.1
≥14	107	23.1
Unknown	67	14.4
Age at first live birth, yr		
<20	69	14.9
20 to 24	94	20.3
25 to 29	76	16.4
≥30	80	17.2
Nulliparous	139	30
Missing data	6	1.3
First-degree relatives with breast or ovarian cancer, *n*		
0	350	75.4
1	101	21.8
>1	13	2.8
Prior breast biopsy, *n*		
0	266	57.3
1	123	26.5
>1	75	16.2
Prior AH		
Yes	20	4.3
No	444	95.7
Gail 5-yr risk estimate, mean ± SD	1.54 ± 1.43	
Gail 5-yr risk estimate, %		
<1.67	309	66.6
≥1.67	155	33.4
Body mass index, kg/m^2^		
<25	182	39.2
25 to 29.9	95	20.5
≥30	108	23.3
Missing data	79	17.0
deCODE genetics panel RR, mean ± SD	1.22 ± 0.44	
<1.00	163	35.1
1.01 to 1.49	182	39.2
≥1.50	119	25.7
Outcome of biopsy		
Benign	366	78.9
AH/LCIS	24	5.2
DCIS	33	7.1
Invasive carcinoma	41	8.8

^a^AH, Atypical hyperplasia; BI-RADS, Breast Imaging-Reporting and Data System; DCIS, Ductal carcinoma *in situ*; LCIS, Lobular carcinoma *in situ*; RR, Relative risk; SD, Standard deviation. Data are number and percent unless otherwise stated.

Table 2 displays the results of age-adjusted and multivariate logistic regression models used to estimate the OR for cancer diagnosis. The SNP panel RR was significantly associated with cancer diagnosis (OR, 2.15; 95% CI, 1.04 to 2.43; *P* = 0.038). The ORs estimated in our model for the categorized SNP panel RRs were comparable to the predefined RR estimates obtained from deCODE genetics. The Gail RR estimate was not significantly associated with cancer diagnosis, nor was Gail absolute 5-year risk ≥1.67%. Among the Gail factors, only age was significantly associated with breast cancer diagnosis, though the ORs for race/ethnicity, age at menarche, age at first live birth and family history of breast cancer were consistent with expected associations. Prior breast biopsy and atypical hyperplasia were inversely associated with breast cancer, though these data were not statistically significant. Few participants (4.3%) reported prior atypical hyperplasia.

**Table 2:** **Logistic regression, odds of cancer among women with BIRADS 4 mammograms,** ***N*** **= 464** ^**a**^

	**Age-adjusted**			**Multivariate** ^**b**^		
	**OR**	**95% CI**	***P*** **-value**	**OR**	**95% CI**	***P*** **-value**
SNP panel RR, log continuous scale	2.15	1.04 to 2.43	0.038	2.30	1.06 to 4.99	0.035
SNP panel RR, categories						
<1.00	1.00	Reference				
1.01 to 1.49	1.09	0.59 to 2.02	0.788			
≥1.50	1.60	0.84 to 3.04	0.149			
Gail RR, log continuous scale	1.11	0.69 to 1.78	0.660			
Gail absolute 5-yr risk, %						
<1.67	1.00	Reference				
≥1.67	1.09	0.60 to 1.98	0.778			
Age, log continuous scale	1.05	1.03 to 1.07	<0.001	1.05	1.03 to 1.08	<0.001
Race/ethnicity						
White	1.00	Reference		1.00	Reference	
African American/black	0.66	0.37 to 1.19	0.170	0.53	0.26 to 1.06	0.071
Other	0.86	0.33 to 2.20	0.748	0.81	0.30 to 2.23	0.689
Age at menarche, yr						
<11	1.44	0.65 to 3.21	0.368	1.33	0.57 to 3.09	0.510
12 to 13	1.67	0.85 to 3.30	0.139	1.50	0.73 to 3.06	0.266
≥14	1.00	Reference		1.00	Reference	
Unknown	0.77	0.29 to 2.07	0.608	0.83	0.28 to 2.41	0.729
Age at first live birth, yr						
<30	1.00	Reference		1.00	Reference	
≥30	1.58	0.81 to 3.08	0.183	1.37	0.66 to 2.87	0.400
Nulliparous	1.09	0.58 to 2.06	0.780	1.06	0.54 to 2.08	0.867
Missing data	1.60	0.17 to 15.0	0.680	2.26	0.22 to 23.6	0.497
First-degree relatives with breast cancer, *n*	1.48	0.86 to 2.57	0.160	1.62	0.90 to 2.90	0.106
Prior breast biopsy	0.73	0.43 to 1.24	0.242	0.82	0.47 to 1.46	0.508
Prior atypical hyperplasia	0.41	0.09 to 1.86	0.247	0.51	0.10 to 2.54	0.410
BMI, kg/m^2^, mean ± SD						
<25	1.00	Reference		1.00	Reference	
25 to 29.9	1.68	0.81 to 3.47	0.161	1.86	0.86 to 4.05	0.116
≥30	1.94	0.99 to 3.81	0.054	2.20	1.05 to 4.58	0.036
Missing data	1.85	0.87 to 3.93	0.111	1.80	0.81 to 3.99	0.147

^a^BI-RADS, Breast Imaging-Reporting and Data System; BMI, Body mass index; CI, Confidence interval; OR, Odds ratio; RR, Relative risk; SD, Standard deviation; SNP, Single-nucleotide polymorphism. ^b^Multivariate model includes log SNP RR, age, BMI, race/ethnicity, age at menarche, age of first birth, family history of breast cancer, breast biopsy and atypical hyperplasia.

In the multivariate model, age, SNP panel RR and BMI were significantly associated with breast cancer diagnosis. Older women were more likely than younger women to be diagnosed with breast cancer (OR = 1.05; 95% CI, 1.03 to 1.08; *P* < 0.001). The SNP panel RR remained strongly associated with breast cancer diagnosis after multivariable adjustment (OR = 2.30; 95% CI, 1.06 to 4.99; *P* = 0.035). Higher BMI was also strongly associated with increased odds of breast cancer diagnosis. Obese women (OR = 2.20; 95% CI, 1.05 to 4.58; *P* = 0.036) had more than twice the odds of cancer diagnosis compared to women with a BMI <25 kg/m^2^.

Next, we evaluated the association of the SNP panel separately for white (*N* = 277) and black (*N* = 145) women (Table 3). Among white women, the SNP panel RR was associated with twofold elevated odds of receiving a cancer diagnosis in both age-adjusted (OR = 2.43; 95% CI, 0.99 to 5.98; *P* = 0.053) and multivariate (OR = 1.97; 95% CI, 0.76 to 5.10; *P* = 0.161) models, and OR estimates were similar for the SNP panel RR categories and predefined values. There was evidence that the SNP panel RR was associated with breast cancer diagnosis among black women. Among black women, the OR estimate was 4.50 in the age-adjusted model (OR = 4.50; 95% CI, 0.87 to 23.2; *P* = 0.073) and 4.21 in the multivariate model adjusted for age, Gail factors and BMI (OR = 4.21; 95% CI, 0.79 to 22.6; *P* = 0.093), though these estimates did not reach statistical significance. In addition, the OR estimates for the SNP panel RR categories were similar to the predefined RR values. There was no significant interaction between race and the SNP panel RR (*P* = 0.880).

**Table 3:** **Logistic regression, odds of cancer among women with BIRADS 4 mammograms, by race** ^**a**^

	**White (** ***N*** **= 277)**				**Black (** ***N*** **= 145)**			
	**Age-adjusted**		**Multivariate** ^**b**^		**Age-adjusted**		**Multivariate** ^**b**^	
	**OR (95% CI)**	***P*** **-value**	**OR (95% CI)**	***P*** **-value**	**OR (95% CI)**	***P*** **-value**	**OR (95% CI)**	***P*** **-value**
SNP panel RR, log continuous scale	2.43 (0.99 to 5.98)	0.053	1.97 (0.76 to 5.10)	0.161	4.50 (0.87 to 23.2)	0.073	4.21 (0.79 to 22.6)	0.093
SNP panel RR, categories								
<1.00	1.00 (reference)				1.00 (reference)			
1.01 to 1.49	1.34 (0.64 to 2.82)	0.437			1.56 (0.90 to 8.18)	0.599		
≥1.50	1.84 (0.83 to 4.11)	0.135			1.77 (0.33 to 9.52)	0.505		

^a^BI-RADS, Breast Imaging-Reporting and Data System; BMI, Body mass index; CI, Confidence interval; OR, Odds ratio; RR, Relative risk; SNP, Single-nucleotide polymorphism. ^b^Multivariate model includes BMI, age, race/ethnicity, age at menarche, age of first birth, family history of breast cancer, breast biopsy, atypical hyperplasia.

We compared the predictive accuracy of the Gail factors, BMI and SNP panel RR (Table 4). First, Gail RR, SNP panel RR and BMI were tested separately in models including age. The model with age and Gail RR had the lowest predictive ability (AUC = 0.6646), and the Hosmer-Lemeshow goodness-of-fit test indicated poor model fit (*P* = 0.0019). All other models exhibited acceptable model fit (*P* > 0.05). The predictive accuracy was similar for age and the SNP panel RR (0.6848) and age and BMI (0.6845). Age, BMI and the SNP panel RR together yielded an AUC of 0.7007, which was of borderline significance compared to age alone (*P* = 0.061).

**Table 4:** **Predictive accuracy of models using Gail risk factors, body mass index and single-nucleotide polymorphism panel among women with BIRADS 4 mammograms** ^**a**^

	**Total study population (** ***N*** **= 464)**				**Age 35 to 49 yr (** ***N*** **= 155)**		**Age ≥50 yr (** ***N*** **= 236)**		**White (** ***N*** **= 277)**		**Black (** ***N*** **= 145)**	
	**AUC**	**GOF** ^**b**^	***P*** **-value** ^**c**^	***P*** **-value** ^**d**^	**AUC**	***P*** **-value** ^**c**^	**AUC**	***P*** **-value** ^**c**^	**AUC**	***P*** **-value** ^**c**^	**AUC**	***P*** **-value** ^**c**^
Age, log Gail RR	0.6646	0.0019	0.839		0.5475	0.966	0.6748	0.470	0.6654	0.373	0.7243	0.495
Age, BMI	0.6845	0.3649	0.210		0.5775	0.619	0.6914	0.613	0.6826	0.317	0.7456	0.653
Age, log SNP RR	0.6848	0.3134	0.197		0.6068	0.445	0.6917	0.508	0.6890	0.153	0.7385	0.836
Age, BMI, log SNP RR	0.7007	0.9297	0.061		0.6258	0.337	0.7086	0.276	0.7078	0.060	0.7527	0.661
Gail factors^e^	0.7144	0.3586	0.044	Reference	0.5488	0.123	0.7115	0.267	0.7390	0.019	0.7256	0.925
Gail factors, BMI	0.7279	0.7646	0.014	0.341	0.6964	0.072	0.7272	0.167	0.7463	0.011	0.7485	0.602
Gail factors, BMI, log SNP RR	0.7377	0.1924	0.007	0.212	0.7242	0.026	0.7356	0.116	0.7518	0.007	0.7719	0.442

^a^AUC, Area under the receiver operating characteristic curve; BI-RADS, Breast Imaging-Reporting and Data System; BMI, Body mass index; RR, Relative risk; SNP, Single-nucleotide polymorphism. ^b^ *P*-value derived from Hosmer-Lemeshow goodness-of-fit (GOF) test. ^c^ *P*-value derived from DeLong test compared to a model with age only. ^d^ *P*-value derived from DeLong test compared to reference model. ^e^Gail factors include age, race/ethnicity, age at menarche, age at first live birth, first-degree family history of breast cancer, breast biopsy and atypical hyperplasia.

Predictive accuracy was greater in the model including the individual Gail risk factors (0.7144) compared to a model with age alone (*P* = 0.044). Adding BMI to the Gail risk factor model increased the AUC (0.7279), but the difference was not statistically significant (*P* = 0.341). Subsequently adding the SNP panel RR to the model further increased the AUC (0.7377; *P* = 0.212). We repeated analyses stratified by age (35 to 49 years and ≥50 years) and found that the addition of BMI and SNP panel RR improved predictive accuracy compared to the Gail factors alone in both age groups, though the AUC values were greater for women ages 50 and older. The addition of the SNP panel had a greater impact on the AUC in the 35 to 49 age group than in women ages 50 and older. When stratified by race, the AUC values were comparable for black women and white women. For the model including Gail factors, BMI and SNP panel RR, the AUC was 0.7518 for white women and 0.7710 for black women. We repeated our analyses excluding women younger than 40, and the results were similar. We performed tenfold cross-validation on the prediction models in the total study population (Table 5). AUC values were slightly attenuated after cross-validation and were not statically significant. The highest cross-validated AUC was observed for the model including age, BMI and the SNP panel (AUC = 0.6753).

**Table 5:** **Cross-validation of prediction models** ^**a**^

	**Total study population (** ***N*** **= 464)**			**Tenfold cross-validation**		
	**AUC**	**95% CI**	***P*** **-value** ^**b**^	**AUC**	**95% CI**	***P*** **-value** ^**b**^
Age, log Gail RR	0.6646	0.5970 to 0.7321	0.839	0.6482	0.5797 to 0.7167	0.159
Age, BMI	0.6845	0.6188 to 0.7501	0.210	0.6583	0.5911 to 0.7255	0.764
Age, log SNP RR	0.6848	0.6195 to 0.7501	0.197	0.6735	0.6077 to 0.7393	0.188
Age, BMI, log SNP RR	0.7007	0.6370 to 0.7645	0.061	0.6753	0.6099 to 0.7407	0.258
Gail factors	0.7144	0.6532 to 0.7755	0.044	0.6522	0.5855 to 0.7188	0.955
Gail factors, BMI	0.7279	0.6705 to 0.7854	0.014	0.6561	0.5919 to 0.7203	0.924
Gail factors, BMI, log SNP RR	0.7377	0.6808 to 0.7946	0.007	0.6727	0.6099 to 0.7356	0.493

^a^AUC, Area under the receiver operating characteristic curve; BMI, Body mass index; CI, Confidence interval; RR, Relative risk; SNP, Single-nucleotide polymorphism. ^b^ *P*-values derived from DeLong test compared to model with age only.

**Figure 1:**
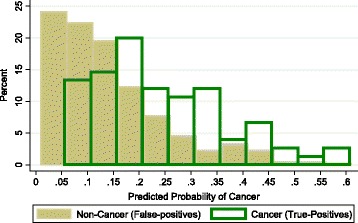
**Distribution of the predicted probability of cancer using Gail factors, body mass index and single-nucleotide polymorphism panel.**

The predicted probabilities of breast cancer diagnosis for each individual were estimated using the model including age, Gail factors, BMI and the SNP panel RR. Figure 1 displays the distribution of predicted probabilities by breast cancer status. Women diagnosed with cancer (true-positives) had a mean predicted probability of cancer diagnosis of 22.6%, compared to 12.2% for women not diagnosed with cancer (false-positives), though the 95% CIs significantly overlapped (Table 6). However, no women diagnosed with cancer had a predicted probability below 5%. On the basis of our model, nine women (3.4%) with BI-RADS 4 mammograms were reclassified below the <2% threshold, none of whom were diagnosed with cancer. Furthermore, 69 women (14.9%) had a predicted probability of cancer less than 5%, and none of these women were subsequently diagnosed with cancer. The positive predictive value of the BIRADS 4 categorization alone was 15.9%, compared to 18.7% using the BIRADS 4 categorization along with the prediction model with a 5% predicted probability.

**Table 6:** **Predicted probability of cancer using Gail factors, body mass index and single-nucleotide polymorphism panel (** ***N*** **= 464)**

		**Cancer**	**Noncancer**
Median % (95% confidence interval)		22.6% (7.0% to 46.6%)	12.2% (2.6% to 38.8%)
Predicted probability, *n* (%)			
	<2%	0 (0%)	9 (1.9%)
	<3%	0 (0%)	35 (7.5%)
	<5%	0 (0%)	69 (14.9%)
	<10%	10 (2.2%)	162 (34.9%)

## Discussion

Our results suggest that breast cancer risk factors can be used to predict cancer diagnosis among women with BI-RADS 4 mammograms. Age, BMI and the 12-SNP panel were strongly associated with cancer diagnosis. Addition of BMI and the 12-SNP panel to Gail risk factors improved model discrimination. Furthermore, using a predicted probability cutoff of 5% for biopsy would reclassify 15% of women below the biopsy threshold while retaining 100% sensitivity in cancer detection in this sample. Though our results need to be prospectively validated, our work provides proof of concept that the use of pretest risk factors to guide follow-up of BI-RADS 4 mammograms could potentially improve mammography screening outcomes by reducing the number of biopsies among women who do not have cancer.

To our knowledge, our present study is the first in which a panel of genetic markers has been tested in women with abnormal mammograms. The SNP panel RR estimates observed were similar to the RR estimates stated by deCODE genetics in our population of women with BI-RADS 4 mammograms, and the SNP panel RR estimate remained strongly associated with cancer diagnosis after adjusting for other breast cancer risk factors. Similar to what has been reported in prior studies [7,8,10-14,44,45], the SNP panel in the present study moderately improved predictive accuracy. However, this small improvement may prove to be more clinically valuable for decisions about biopsies among women with abnormal mammograms than for risk stratification in the general population.

It was not entirely surprising that the Gail risk estimate was not significantly associated with cancer diagnosis in our study, because the Gail model was developed to estimate 5-year or lifetime risk of invasive breast cancer in the general population. In our present study, we attempted to predict the risk of diagnosis of either DCIS or invasive cancer in women with abnormal mammograms. The magnitudes of the exposure–disease relationships are likely different for short-term cancer outcomes in the higher-risk BI-RADS 4 population. In our analysis, the model using age and the Gail RR had poor calibration, and therefore the AUC estimates are not meaningful. The poor calibration of this model could have been due to differences in the study population and outcome used in our study, or it could have been a result of our inclusion of age as a continuous predictor to provide a more parsimonious model, whereas the original Gail model used 5-year age categories. Because of this, we also examined a model that entered all Gail risk factors individually, and this model was better calibrated to our data. We observed an AUC of 0.738 for the model with Gail factors, BMI and the SNP panel, which is higher than the AUC observed in the general population for the Gail model alone (0.596) or the Gail model including breast density (0.634) [46]. Researchers in two prior studies evaluated prediction models in women with BI-RADS 4 mammograms. A prediction model trained on 170 French patients with BI-RADS 4 mammograms using Gail risk, age, presence of a palpable lesion, lesion size, hormone replacement therapy and menopause status demonstrated predictive accuracy similar to our model, with an AUC of 0.716 in the training set and AUC of 0.660 when validated in 188 BI-RADS 4 patients from Texas [47]. Similar to our results, age was the strongest predictor of cancer among approximately 4,000 women with BI-RADS 4 mammograms referred for biopsy between 1997 and 2001 in the Vermont Breast Cancer Surveillance System [48]. The presence of a palpable lump, previous breast biopsy, menopause status and use of postmenopausal hormone therapy were also associated with cancer diagnosis. Genetic risk factors and BMI were not included in these prediction models.

Obese women had more than twice the odds of receiving a cancer diagnosis compared to women of normal weight. One possible explanation for this association is that obese women tend to have less-dense breasts and therefore potentially easier-to-read mammograms, which facilitates a more accurate interpretation of their mammograms by radiologists, such that obese women with a BI-RADS 4 mammogram are more likely to actually have cancer (and less likely to have a false-positive test) than nonobese women. The association of BMI with cancer diagnosis may also reflect disease etiology, as BMI is associated with increased risk of postmenopausal breast cancer [49]. Although BMI data were missing for 17% of participants, we do not believe the missing data biased the observed association. The distribution of risk factors (except for age at first birth) and percentage diagnosed with cancer did not differ for women with missing BMI data and women with complete BMI data. Additional studies are needed to verify this association and to tease apart the effects of BMI and breast density in women with abnormal mammograms.

This study was a first attempt to validate the 12-SNP panel among black women. The SNP panel variants were identified and validated primarily in white/European populations. Several genome-wide association studies and candidate gene studies [50-60], and authors of meta-analyses [61-66] have assessed the association of these 12 SNPs individually with breast cancer risk among black/African American populations, with mixed results. Six of the twelve SNPs in the panel have been replicated in at least one study of black/African American populations: rs1045485 (CASP8) [59], rs1219648 (FGFR2) [54,58,59], rs13387042 (2q35) [52,58,59], rs3817198 (LSP1) [60], rs4415084 (FGF10) [56] and rs999737 (14q24.1, RAD51B) [59]. Validating breast cancer–associated SNPs among black women is challenging, given the large sample sizes needed to detect small associations, differing linkage disequilibrium patterns among different ancestral groups, and disease heterogeneity. Despite the fact that only half of these SNP associations have been replicated, the 12-SNP panel appeared to have predictive value among black women, though our results need to be validated in larger studies. In addition, in future studies, researchers should assess whether race-specific and tumor subtype–specific SNP panels can further improve breast cancer risk prediction.

Several limitations should be considered when interpreting our results. Because we recruited women referred for biopsy at one academic hospital, our study sample may not be representative of all women with abnormal mammograms referred for biopsy. Our sample size was modest, and therefore our results, particularly those of subgroup analyses, should be interpreted cautiously. We performed cross-validation of our prediction models for the entire study sample; however, prospective validation of our results is needed. Given the limited number of cancers (*N* = 75), our study did not have statistical power to fit separate models for DCIS and invasive cancer or to assess interactions between risk factors. We utilized a validated panel of 12 breast cancer–associated SNPs. To date, nearly 70 SNPs have been identified that are associated with breast cancer risk [67]. Therefore, our results using 12 SNPs may underestimate the utility of genetic markers, and including a larger number of genetic markers may further improve risk prediction. In future studies, researchers should evaluate the use of genetic markers in women with abnormal mammograms. Also, breast density was not controlled for, and this may partly explain the observed association of BMI with cancer diagnosis.

This study has several strengths. Ours is one of the first studies to develop a cancer prediction model for women with abnormal mammograms. We had rich data on recognized breast cancer risk factors ascertained prior to biopsy. We employed a validated panel of genetic markers associated with breast cancer incidence, with RR estimates independent of traditional breast cancer risk factors. Our study population was diverse in terms of age and race/ethnicity, suggesting that our model could be applied broadly.

## Conclusions

Our results suggest that pretest breast cancer risk factors could be utilized to individualize biopsy decisions following abnormal mammograms. We found that age, BMI and a 12-SNP panel were significantly associated with breast cancer diagnosis in women with BI-RADS 4 mammograms. The association of obesity with cancer diagnosis was particularly novel and warrants additional investigation. On the basis of results derived from the model using Gail risk factors, BMI and genetic markers, we were able to identify a predicted probability threshold that could be used to identify women who would not benefit from immediate biopsy. Our study, though preliminary, highlights that improved risk modeling for women with abnormal mammograms could reduce the burden of false-positive tests and therefore increase the benefits of mammography. Future studies are needed to validate these results in larger patient populations.

## Additional file

Additional file 1:
**Expected and observed allele frequencies (** ***N*** **= 464).**

## Abbreviations

AH: Atypical hyperplasia
AUC: Area under the receiver operating characteristic curve
BCRAT: Breast Cancer Risk Assessment Tool
BI-RADS: Breast Imaging-Reporting and Data System
BMI: Body mass index
CI: Confidence interval
DCIS: Ductal carcinoma *in situ*
LCIS: Lobular carcinoma *in situ*
OR: Odds ratio
RR: Relative risk
SD: Standard deviation
SNP: Single-nucleotide polymorphism
