# Supplementary material for: The use of the Gail model, body mass index and SNPs to predict breast cancer among women with abnormal (BI-RADS 4) mammograms
Source: Breast Cancer Res. 2015 Jan 8;17(1):1. doi: 10.1186/s13058-014-0509-4 (PMC4311477; doi:10.1186/s13058-014-0509-4)
Supplement: Additional file 1: — Expected and observed allele frequencies ( N = 464). [file 13058_2014_509_MOESM1_ESM.docx]

| **Additional file 1: Expected and Observed Allele frequencies (N=464)** | | | | | |
| --- | --- | --- | --- | --- | --- |
| **SNP** | **Risk Allele** | **Homozygote Relative Risk** | **Expected Allele Frequency** | **Observed Allele Frequency** | **p-value** |
|  |  |  |  |  |  |
| rs1045485 (CASP8) | G | 1.03 | 0.870 | 0.901 | 0.047 |
|  |  |  |  |  |  |
| rs11249433 (1p11.2) | C | 1.18 | 0.390 | 0.297 | <0.001 |
|  |  |  |  |  |  |
| rs1219648 (10q26, FGFR2) | G | 1.31 | 0.380 | 0.433 | 0.019 |
|  |  |  |  |  |  |
| rs13281615 (8q24.21) | G | 1.10 | 0.400 | 0.462 | 0.006 |
|  |  |  |  |  |  |
| rs13387042 (2q35) | A | 1.19 | 0.497 | 0.612 | <0.001 |
|  |  |  |  |  |  |
| rs3803662 (16q12, TOX3) | T | 1.42 | 0.269 | 0.421 | <0.001 |
|  |  |  |  |  |  |
| rs3817198 (11p15, LSP1) | C | 1.10 | 0.300 | 0.294 | 0.778 |
|  |  |  |  |  |  |
| rs4415084 (5p12, FGF10) | T | 1.19 | 0.396 | 0.499 | <0.001 |
|  |  |  |  |  |  |
| rs4973768 (3p24, SLC4A7) | T | 1.12 | 0.460 | 0.459 | 0.966 |
|  |  |  |  |  |  |
| rs6504950 (17q23.2, STXBP4) | G | 1.03 | 0.720 | 0.705 | 0.472 |
|  |  |  |  |  |  |
| rs889312 (5q11.2, MAP3K1) | C | 1.19 | 0.280 | 0.313 | 0.113 |
|  |  |  |  |  |  |
| rs999737 (14q24.1, RAD51B) | C | 1.06 | 0.760 | 0.842 | <0.001 |
|  |  |  |  |  |  |
| * p-value from one-sample t-test comparing observed to expected frequency | | | |  |  |
